# Relationships and Communication—the core components of person‐centred care

**DOI:** 10.1111/hex.13153

**Published:** 2020-11-28

**Authors:** Kerry Kuluski

**Affiliations:** ^1^ Institute for Better Health Trillium Health Partners Ontario Canada; ^2^ Institute of Health Policy, Management and Evaluation University of Toronto Toronto Canada

What makes a health‐care experience good? In addition to receiving timely access to treatment and care, the quality of relationships that people have with health and social care providers is an essential component,^1^, ^2^, ^3^ particularly when managing multiple, complex health problems.^4^, ^5^

In this issue of *Health Expectations, there is a variety of papers that provide insight into the experiences of people with complex care needs, including populations that have been previously understudied and overlooked*. A recurring theme is the importance of clear and ongoing communication, preparing for next steps in the illness journey, trust in the care provider and team and the importance of social health (identity and roles, not just disease profiles).

For example, in Desborough et al's^6^ meta‐synthesis of qualitative studies of experiences of people with multiple sclerosis (MS)—core themes included: a need for knowledge; uncertainty; loss of roles; threats to a changing identity; managing fatigue and relationships; and adapting to life with the disease. These themes capture core components of care that can inform care delivery and are likely transferrable to other populations with complex, degenerative conditions. Some similarities were noted in Pétrin et al's^7^ paper on people with MS in Canada where care avoidance occurred due to the tiresome and onerous process of accessing care (which was perceived to outweigh the benefits of receiving care).

Akanuwe et al^8^ conducted the first study of the lived experience of people with Guillain‐Barre syndrome in the UK reporting the key needs across the illness journey—the importance of early diagnosis, enhanced communication, information from health‐care staff and looking to the future to achieve improved function.

Willis et al^9^ examined the experiences of older trans‐identifying adults who were seeking trans‐related medical care while they were in the process of transitioning medically. Participant accounts revealed wide variations in the general practitioners (GPs) knowledge regarding their needs. Participants felt that the responsibility was on them to educate their GPs on care options. Some of the participants experienced discriminatory responses from health‐care professionals. The journey in receiving gender affirming treatments was characterized by many delays and much uncertainty.

Other papers in this collection provide insight on *how to effectively engage people in their care*.

Tolvanen et al^10^ conducted a comparative analysis of factors associated with patient enablement in primary care across 31 countries using multi‐level logistic regression models. Patient enablement was defined as the ability to understand and cope with illness and life after a consultation with a doctor. Most strongly associated with enablement were patient‐level factors (eg older age, female) as well as perceptions of the consultation (eg more trust in the GP, greater continuity of care). Patients from long‐term oriented cultures (oriented towards preparing for the future) had a decreased risk of lower enablement.

Abdullah et al's^11^ paper focused on a key component that impacts on patient engagement—health literacy (the ability to access, understand and appraise health information) within a multi‐ethnic Asian population with type 2 diabetes. They found variability of healthy literacy among the patients studied. While some patients actively sought out and appraised information, others relied on others they trusted (including health‐care providers and family) and accepted guidance or treatment, without further evaluation. The term, ‘distributed health literacy’, was used by the authors demonstrating the important role of social networks in supporting patients in their understanding and management of illness.

*Ideas on how to ‘activate’ engagement in care is provided in other papers in this collection*.

For example, Lindig et al^12^ translated the Ask 3 Questions (Ask3Q) intervention into German and studied its acceptability and feasibility among patients and health‐care professionals. The three questions: *what are my options? what are the benefits and harms? and how likely are these?,* were perceived by participants as a feasible tool to empower patients to ask more questions as well as a reminder for physicians to convey important information.

Salmi et al^13^ proposed a new framework to conduct case‐based clinical reviews to guide clinical decisions called ‘Shared Decision Evidence Summary’ (SHaDES). SHaDES combines relevant scientific evidence, psychosocial components and patient's perspectives to guide treatment decision making. SHaDES addresses the limits of evidence‐based medicine and clinical practice guidelines which are often criticized for having a narrow medical focus and for excluding patient preferences.

The benefits of shared decision making (SDM) with surgical patients were found in a scoping review by Niburski et al.^14^ SDM is a model of collaboration where patients and their providers work together to determine a treatment or care plan that reflects their values and preferences. The scoping review findings indicated that the use of shared DM decreased the surgical intervention rate, decisional conflict, decision regret and improved trust.

These tools and strategies provide mechanisms to gain a deeper understanding of patients and their personal needs. The paper by Goodrich et al^15^ took this to a deeper level through a study that consisted of care providers shadowing patients at the end of life. This experiential technique was intended to help care providers better understand the patients experience and point of view. In their study, they wanted to explore acceptability among clinical and non‐clinical staff and examine how it motivated them to make improvements in care. For many providers, it was a deeply emotional experience and influenced their motivation to improve patient care while patients and families welcomed the additional care and attention.

This editorial briefing provides a sample of the thought‐provoking papers that are published in this issue of Health Expectations. A deep understanding of the lived experience of people with a variety of health and social care needs from a variety of cultural contexts; along with insights on factors that influence engagement; and tools and approaches to improve experience provides important evidence to inform clinical care and policy worldwide.
